# Attenuation of *Vibrio parahaemolyticus* Virulence Factors by a Mixture of Natural Antimicrobials

**DOI:** 10.3390/microorganisms7120679

**Published:** 2019-12-11

**Authors:** Laurette Pinkerton, Mark Linton, Carmel Kelly, Patrick Ward, Gratiela Gradisteanu Pircalabioru, Ioan Pet, Lavinia Stef, Filip Sima, Tabita Adamov, Ozan Gundogdu, Nicolae Corcionivoschi

**Affiliations:** 1 Bacteriology Branch, Veterinary Sciences Division, Agri-Food and Biosciences Institute, Belfast BT9 5PX, UK; laurette.pinkerton@afbini.gov.uk (L.P.); mark.linton@afbini.gov.uk (M.L.); carmel.kelly@afbini.gov.uk (C.K.); filip.sima@afbini.gov.uk (F.S.); 2 Auranta, NovaUCD, Dublin 4, Ireland; pat@auranta.ie; 3 Research Institute of University of Bucharest, 300645 Bucharest, Romania; gratiela87@gmail.com; 4 Banat University of Animal Sciences and Veterinary Medicine - King Michael I of Romania, 300645 Timisoara, Romania; ioanpet@eurofins.com (I.P.); Lavi_stef@animalsci-tm.ro (L.S.); tabitaadamov2003@yahoo.com (T.A.); 5 London School of Hygiene and Tropical Medicine, London WC1E 7HT, UK

**Keywords:** *Vibrio parahaemolyticus*, virulence, natural antimicrobials

## Abstract

Reducing acute mortality in aquatic crustaceans using natural alternatives to antibiotics has become a necessity, firstly for its positive impact on the aquaculture industry and, secondly, because the extensive use of antibiotics may lead to increased levels of drug resistance in pathogenic microorganisms. This study aimed to investigate the effect of a mixture of natural antimicrobials on the in vitro and in vivo virulence abilities of Type VI secretion system (T6SS)-positive *Vibrio parahaemolyticus* (A3 and D4), strains known as having potentially harmful health consequences for aquatic crustaceans and consumers. Herein, we report that a natural antimicrobial mixture (A3009) was capable of significantly reducing the virulence of *V. parahaemolyticus* strains A3 and D4 in an in vitro infection model, using the fish cell line CHSE-214, an effect which correlates with the bacterial downregulation of *hcp*1 and *hcp*2 gene expression and with the ability of the antimicrobial to efficiently retain low cytotoxic levels (*p* < 0.001). We show for the first time that a natural antimicrobial is able to significantly reduce the mortality of shrimps in a challenge experiment and is able to significantly attenuate H_2_O_2_ release during infection (*p* < 0.001), indicating that it could harbor positive intestinal redox balance effects.

## 1. Introduction

*V. parahaemolyticus* is a Gram-negative bacterium, halophilic in nature with a typically rigid curve or straight non-spore forming rod shape [1]. The bacteria of the *Vibrio* genus are commonly recognised as inhabitants of fresh and marine waters, with over 100 species being described so far [2]. Bacterial pathogens commonly elicit an inflammatory response upon infection; however, *V. parahaemolyticus* has not been shown to do so [3]. *V. parahaemolyticus* is a toxin producing bacterium, in which the toxins are controlled by the genes *pirA* and *pirB* and together are responsible for acute hepatopancreatic necrosis disease (AHPND) [4]. Individually, the toxins appear to be incapable of inducing AHPND and both genes must be present for induction to take place [5]. This syndrome has proven to be a frequent cause of early mortality syndrome (EMS) in the US and many Asian countries [6]. AHPND induces severe atrophy of aquatic crustaceans [7] and has been responsible for considerable aquaculture production losses [8]. Studies have endeavoured to determine the causative agent, collectively identifying the primary pathogen as *V. parahaemolyticus* [3].

The Type VI secretion systems (T6SS) are associated with severe forms of gastrointestinal diseases and have been described mostly in Gram-negative bacteria [9]. Bacteria use these secretion systems to eliminate competing microorganisms by transporting toxins into target bacteria or eukaryotic cells, leading to a reduction in growth and ultimately death [10]. Coincidentally, the T6SS was first discovered in *Vibrio cholerae* [11] and it was characterised by being responsible for the secretion of haemolysin protein (*hcp*), an important virulence factor in many bacteria, including *Pseudomonas aeruginosa* [12]. Infecting human gastrointestinal cells lines (HCT-8) with *Campylobacter* spp. highlighted that the T6SS system is involved via the downregulation of *hcp* gene expression in the reduction of the bacterium’s ability to adhere and infect in vitro [13]. The same study also showed that the effect was extrapolated in vivo when chicken broilers where challenged with *Campylobacter* spp. In *V. parahaemolyticus*, the *hcp* haemolysin signature gene has two proteins, hcp1 and hcp2, which interestingly are expressed under different growth conditions mainly related to salt concentration (1.5–3% NaCl) [14]. The differential expression of *hcp*1 and *hcp*2 and their dependency on the salt concentration seems to be related to the ability of the pathogen to compete for a niche, especially in a marine environment, and to enhance their virulence ability [14].

*V. parahaemolyticus* detection strategies are continually being developed in an attempt to reduce the risk of infection via seafood consumption [15,16]. Various other control and prevention measures have also been proposed to limit the growth of *V. parahaemolyticus* in contaminated seafood and prevent potential outbreaks [17]. However, due to its ubiquitous nature in marine environments and relatively low infectious dose, bacteria-free seafood is seemingly unattainable [18]. Antibiotics such as tetracycline are used extensively in aquaculture in an attempt to improve food safety [19]. Despite their efficiency and low cost, antibiotic overuse has become associated with increasing levels of drug resistance and the establishment of resistance determinants [20]. The large-scale disinfection of water prior to stocking is a current practice to remove potential pathogens. This practice causes a considerable ecosystem disturbance by increasing the nutrient availability post-disinfection; destabilising the microbial community and in turn encouraging environmental re-colonisation with fast-growing bacteria, such as the pathogenic *Vibrio* species [21]. 

This study aimed to investigate how a mixture of natural antimicrobials could regulate the growth of *V. parahaemolyticus* strains A3 and D4 in vitro and in vivo and identify the virulence factors involved. We also aimed to investigate their potential to attenuate *V. parahaemolyticus* virulence by using sub-lethal concentrations of the antimicrobial mixture as there is a constant concern that bacteria could also gain resistance to natural antimicrobials [22].

## 2. Materials and Methods 

### 2.1. Bacterial and Eukaryotic Cells Lines and Antimicrobials

The *Vibrio parahaemolyticus* A3 (origin Vietnam) and D4 (origin Mexico) strains were kindly donated by Kim Orth from the Department of Molecular Biology, University of Texas Southwestern Medical Center, Dallas, TX, USA. Both strains were routinely cultured in marine Luria–Bertani (MLB) broth (Luria–Bertani broth containing 1.5% or 3.5% NaCl). The CHSE-214 cells were obtained from the European Collection of Authenticated Cell Cultures (ECACC). The CHSE-214 cells (ECACC No. 91041114) are a fish cell line derived from *Oncorhync hustshawytscha* embryos. They were cultured in minimum essential medium (MEM) (Gibco, Oxford, UK) supplemented with 10% foetal bovine serum (Life Technologies, Oxford, UK), 2 nM l-glutamine (Corning, Oxford, UK), and an antibiotic/antimycotic solution (10,000 IU/mL penicillin, 10,000 μg/mL streptomycin and 25 μg/mL amphotericin B). A mixture of antimicrobials containing lactic acid, E330 citric acid and citrus extract was used. The composition (per kg) of the feed material was sodium chloride (1 g) and maltodextrin. The additives (per kg) were preservatives, including lactic acid and E330 citric acid, and the flavouring was citrus extract. The analytical constituents (%) were crude ash (0.2%), crude protein (0.84%), crude oils and fats (10.83%), crude fibre (0.03%), phosphorus (0.01%), sodium (1.12%), calcium (1.34%), lysine (0%) and methionine (0%). The antimicrobial product (Auranta 3009, Nova UCD, Ireland) was obtained from Envirotech Innovative Products Ltd. 

### 2.2. Determination of Minimum Inhibitory Concentrations

The minimum inhibitory concentration (MIC) and minimum microbicidal concentration (MBC) were determined for two strains of acute hepatopancreatic necrosis disease (AHPND)-causing *Vibrio parahaemolyticus*, strains A3 and D4. The strains were grown overnight at 37 °C in nutrient broth (Oxoid, Basingstoke, UK) containing 1.5% or 3% NaCl. The resulting stationary phase cultures were diluted using a nutrient broth containing 1.5% or 3% NaCl, to give a suspension containing approximately 6 log CFU/mL A series of Auranta 3009 solutions were prepared in a nutrient broth containing 1.5% or 3% NaCl, to give a range of concentrations from 4% down to 0.015625%. One ml of each Auranta 3009 solution was transferred into separate sterile plastic bijou bottles and 1 mL of either strain of *V. parahaemolyticus* was added to each bottle. A positive control containing 1 mL of nutrient broth and 1 mL of the 6 log CFU/mL suspension was prepared. The negative control was 2 mL of uninoculated nutrient broth plus salt. The mixtures were incubated aerobically at 37 °C for 24 h. This procedure was repeated on three separate occasions. The MIC value was determined as the lowest concentration of Auranta 3009 that showed no bacterial growth. After 24 h, the bijou bottles were observed for bacterial growth. If the broth was clear, indicating no bacterial growth, then 100 µL was spread-plated onto nutrient agar containing 1.5% or 3% NaCl using a sterile glass spreader. This was repeated for all concentrations of Auranta 3009 that appeared clear in the bijou container. All plates were incubated aerobically at 37 °C for 24 h. This procedure was repeated on three separate occasions for both *V. parahaemolyticus* strains A3 and D4. The MBC value was determined as the lowest concentration of Auranta 3009 that showed no bacterial growth on the plates.

### 2.3. Growth Curves

Growth curves were established for both strains of *V. parahaemolyticus*; A3 and D4. Broth cultures were prepared in a nutrient broth containing 1.5% or 3% NaCl and incubated overnight at 37 °C. These were diluted using nutrient broth containing 1.5% or 3% NaCl, to give a final concentration of approximately 6 log CFU/mL. The MIC value for both *V. parahaemolyticus* A3 and *V. parahaemolyticus* D4 was previously determined to be 0.0625% of Auranta 3009. An Auranta 3009 solution was prepared using a nutrient broth containing 1.5% or 3% NaCl to give a final concentration of 0.0625% when the suspension of bacteria was added. Two-fold dilutions of this solution were prepared in a 96-well plate giving a range of Auranta 3009 concentrations from the MIC (0.0625%) down to 1/8 of the MIC concentration (0.0078125%). The final volume of Auranta 3009 solution in each well was 90 µL. The bacterial suspension (10 µL) was added to each well and thoroughly mixed. Nutrient broth containing 1.5% or 3% NaCl with no Auranta 3009 added was inoculated with the bacterial suspension as a positive control. The negative control was uninoculated nutrient broth containing 1.5% or 3% NaCl. The 96-well plate was sealed with plastic film and the optical density was measured at 600 nm at intervals of 1 h over a 24 h period at 37 °C using a FLUOstar Omega automatic plate reader (BGM Labtech, Aylesbury, UK). This procedure was repeated on three separate occasions for both *Vibrio parahaemolyticus* A3 and D4.

### 2.4. Challenge Tests (Counting Living Larvae)

*V. parahaemolyticus* strains A3 and D4 were selected to be tested for their pathogenicity by a challenge test using healthy *Penaeus vannamei* post larvae, following a procedure previously described [23]. Twenty-five shrimp post larvae per replicate were plated in sterile petri dishes and exposed to infection with 10^9^/mL bacteria for 10 min. The antimicrobial mixture was applied at the time of infection in concentrations of 0.0625%, 0.031%, 0.015% and 0.007% in 500 mL flasks. Survival was determined by counting the larvae at 30 h after infection. A positive and a negative control (± antimicrobial mixture or ± larvae) was also included in the challenge at 0% of the antimicrobial mixture. The experiment was performed in triplicate. 

### 2.5. H_2_O_2_ Production in CHSE-214 Cells Response to Treatment Following Infection

A culture mixture of *V. parahaemolyticus* strains A3 and D4 was prepared as described above. The CHSE-214 cells were grown in a minimum essential medium (Minimum Essential Media (MEM) -Gibco^®^, Waltham, MA, USA) until they reached confluence and were supplemented with 10% fetal bovine serum and 2 mL glutamine. The cells were routinely grown in 75 cm^2^ tissue culture flasks (Sigma-Aldrich, St. Louis, MO, USA), in a humidified incubator at 37 °C with 5% CO_2_. The H_2_O_2_ production from infected and un-infected CHSE-214 in response to treatment with Auranta 3009 at was measured using the PeroxiDetect™ Kit (Sigma-Aldrich, St. Louis, MO, USA), following manufacturer guidelines and as described in [24]. 

### 2.6. In Vitro Adhesion and Infection Assay on CHSE-214 Cell Line and qRT-PCR for hcp1 and hcp2 Expression

To confirm the efficacy of Auranta 3009 in reducing the adhesion and invasion abilities of *V. parahaeomolyticus* strains A3 and D4 the isolates were grown to the OD_600_ of 0.5. The CHSE-214 were cultured as described above. Prior to the infection of the cells, the cell monolayers were incubated for 3 h with tissue culture media containing 0.031% Auranta 3009 followed by washing three times with the tissue culture media, and then infected for 3 h with 10^4^ CFU/mL *V. parahaemolyticus* strains A3 and D4. After infection, the infection media was removed and the infected monolayers were washed three times with the tissue culture media. The infected cells were then incubated with tissue culture media containing gentamicin (100 µg/mL) in order to expose the internalised bacteria or to total lysis without gentamicin inclusion, but, instead, 0.1% Triton X was included to reveal the total bacterial adhesion. In order to determine the role of *hcp*1 in marine conditions (3% NaCl), in *V. parahaeomolyticus* infection, we have co-cultured the microorganism in nutrient broth containing 1.5% or 3% NaCl in the presence of 0.031% Auranta 3009 for 3 h. The co-cultured microorganisms were then used in the infection assay at an MOI of 100. Similarly, the experiment was also performed to investigate the effect of *hcp2* expression, but this time with bacteria grown in low salt concentrations (1.5% NaCl). The bacteria grown in the above conditions was also used to determine the *hcp1* and *hcp2* gene expression in the above conditions. The primers used to detect *hcp1* were: cacgtgacggctcggtgg and ctcttctttcgcgtcttggtcg and for *hcp2* cgagtatccactcgaaactttc and ttctgctccctcagtactttctg [14]. The RNA was reverse transcribed using Transcriptor First Strand cDNA Synthesis Kit (Roche, United Kingdom) according to the manufacturer’s protocols. The mRNA levels were determined by quantitative RT-PCR using QuantiNovaSYBR^®^ Green PCR Kit (Qiagen, Manchester, UK) on a LightCycler^®^ 96 (Roche, Buckinghamshire, UK). A total of 5 μL of SYBR Green master mixture was used in each reaction, along with 0.5 μL of 10 μM primer mixture, 3 μL of molecular grade water, and 1 μL of DNA sample. For *hcp* 1 and 2 (2 min at 95 °C, followed by 40 cycles of 95 °C for 5 s, 60 °C for 10 s, and a final extension at 72 °C for 5 min), a total of 5 μL of SYBR Green master mixture was used in each reaction along with 0.8 μL of 20 μM primer mixture, 7.4 μL of molecular grade water, and 1 μL of DNA sample. The relative quantity of the mRNA was calculated using the ΔCt method. The *rARN 16S* gene was used as an endogenous control since it was transcribed in equal rates in both the treated and untreated cells.

### 2.7. Cytotoxicity Assay

The CHSE-214 cells were infected as described above over a 4 h time course with *V. parahaemolyticus* strains A3 and D4. The direct impact of Auranta 3009 was investigated by pre-treatment of CHSE-214 cells with 0.031% Auranta 3009 for 3 h. After 3 h, the media was removed, the cells were washed 3× with fresh MEM, followed by infection with *V. parahaemolyticus* strains A3 and D4 at an MOI of 100. The CHSE-214 cells were also infected with pre-treated bacteria. Uninfected cells exposed to 0.031% of Auranta 3009 were used as controls. Lactate dehydrogenase release (LDH) was measured with a cytotoxicity detection kit (Roche, Buckinghamshire, UK) following the manufacturer’s instructions. The results are expressed as cytotoxicity calculated as a percentage of the total lysis of cells lysed in Triton X 100.

### 2.8. Motility Assay 

Motility was assessed as previously described [25]. Briefly, cultures of *V. parahaemolyticus* strains A3 and D4 were co-cultured in nutrient broth containing 1.5% NaCl or 3% NaCl in the presence of 0.031% Auranta 3009 for 3 h. The co-cultured organisms where then placed (~1 × 10^6^ CFU) at the centre of the agar plate. The diameter of the motility area was measured. Non-co-cultured strains were used as a control. The experiments were performed in triplicate and on three separate occasions. 

## 3. Results

### 3.1. The Role of Antimicrobial Mixtures in the Growth and Survival of V. parahaemolyticus Strains A3 and D4

**Figure 1:**
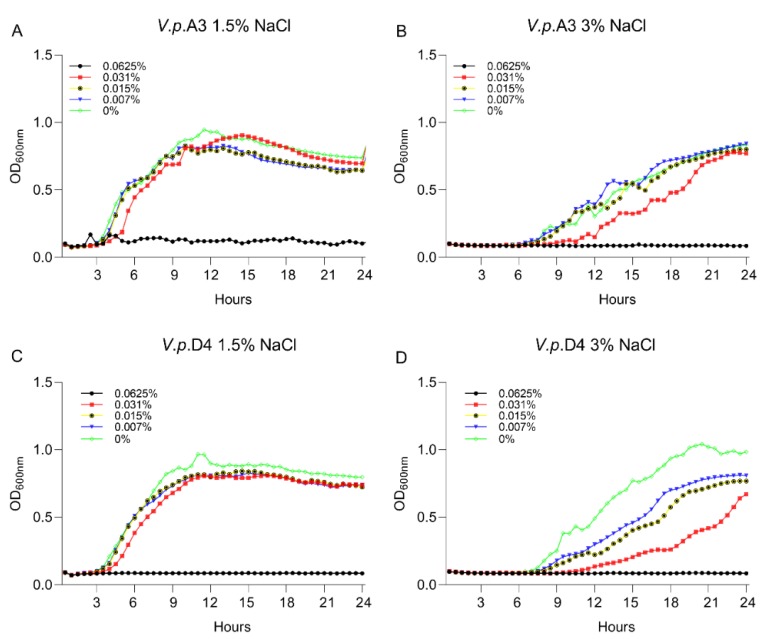
The impact of A3009 on the growth and survival of *Vibrio parahaemolyticus* A3 (**A**)—1.5% NaCl, (**B**)—3% NaCl and D4 (**C**)—1.5% NaCl, (**D**)—3% NaCl. The MIC concentrations are indicated on the graphs. The experiments were performed in triplicate and on three separate occasions. In order to quantify the growth, the absorbance was measured at 600 nm every 0.5 h for 24 h.

First, we aimed to gain insight into the growth characteristics of *V. parahaemolyticus* strains A3 and D4 in the presence of 1.5% and 3% NaCl. The experiment was designed to identify the concentration at which the antimicrobial mixture has no lethal effect on the *V. parahaemolyticus* strains A3 and D4. This concentration was required to allow us to investigate the possible alteration of bacterial virulence factors while the pathogens are still alive. The MIC and MBC values at levels of 1.5% and 3% NaCl against *V. parahaemolyticus* A3 and *V. parahaemolyticus* D4 are shown in Table 1. In order to identify the effect of the antimicrobial mixture on the bacterium virulence factors, we used various concentrations and identified that at half ratio (1/2) of the highest MBC (0.031%), the antimicrobial mixture had no lethal effect; however, it disrupted the growth of both strains (Figure 1). Firstly, we have concluded that the salt concentration had no significant effect on the MIC and MBC values and, secondly, that the concentration of 0.031% of the antimicrobial mixture can be used further in our investigations to assess the implications in virulence.

### 3.2. Motility

**Figure 2:**
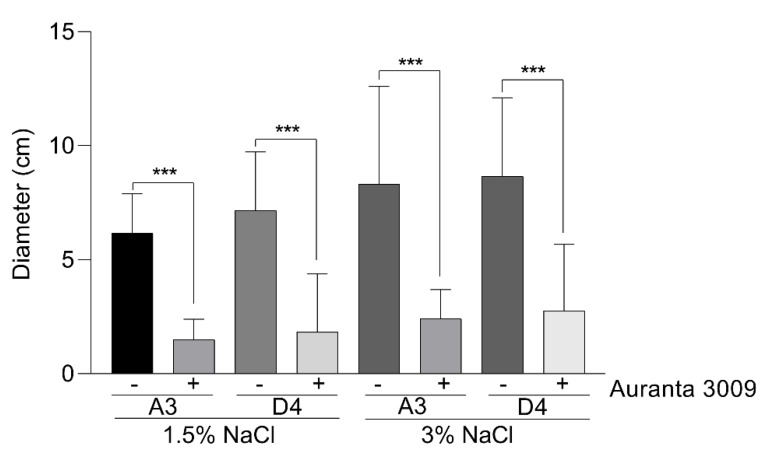
The effect of 0.031% (concentration) Auranta 3009 on *V. parahaemolyticus* A3 and D4 motility. Asterisks indicate significant differences (∗∗∗ *p* < 0.001). Error bars represent the standard deviation of means from three different experiments, each experiment being performed in triplicate.

We next investigated the potential impact of the antimicrobial mixture on the *V. parahaemolyticus* A3 and D4 motility, a major virulence factor, at the sub-inhibitory concentration calculated at half of the MIC (0.031%) in the presence of 1.5% and 3% NaCl (Figure 2). The motility plate assays clearly showed that both strains are generally very motile, but the pre-treatment with 0.031% Auranta 3009 significantly reduced motility, independent of the NaCl_2_ concentration (∗∗∗ *p* < 0.001). These results suggested that the antimicrobial mixture reduces the bacterial motility and potentially has an impact on virulence.

### 3.3. The Impact of Auranta 3009 on the hcp1 and hcp2 Gene Expression at 1.5% and 3% NaCl

**Figure 3:**
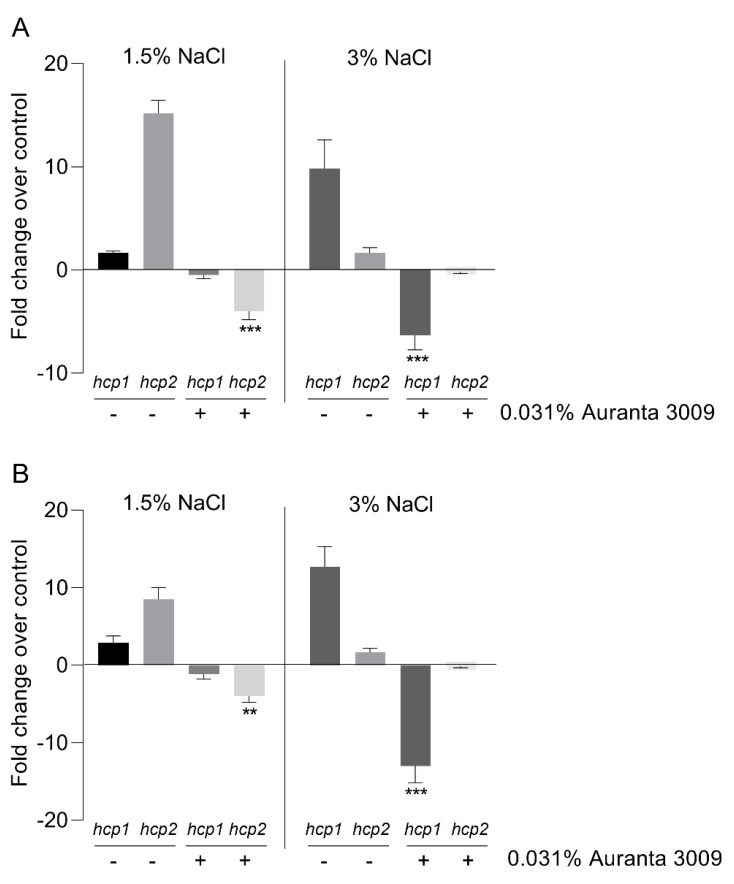
Effect of the antimicrobial mixture Auranta 3009 on *V. parahaemolyticus* A3 and D4 *hcp*1 *and hcp2* gene expression in the presence of 1.5% and 3% NaCl. *V. parahaemolyticus* A3 (**A**) and D4 (**B**). Asterisks indicate significant differences (Student’s *t*-test ∗∗ *p* < 0.01, ∗∗∗ *p* < 0.001). Error bars represent the standard deviation of means from three different experiments performed in triplicate.

The effect of the antimicrobial mixture on *V. parahaemolyticus* A3 and D4 *hcp* gene expression is shown in Figure 3. The qRT-PCR results indicate a significant downregulation (∗∗∗ *p* < 0.001) in *hcp*1 gene expression levels in the presence of 3% NaCl in *V. parahaemolyticus* A3, with no effect on *hcp*2 at this salt concentration. The downregulation effect on *hcp*2 was only observed in the presence of 1.5% NaCl, as indicated in Figure 3 (panel A) (∗∗∗ *p* < 0.001). The results were similar for *V. parahaemolyticus* D4 in regard to the downregulation effect on *hcp*1 (∗∗ *p* < 0.01) and *hcp*2 (∗∗∗ *p* < 0.001) gene expression produced by Auranta 3009 at 1.5% and 3% NaCl (Figure 3, panel B). 

### 3.4. Infection Assay

**Figure 4:**
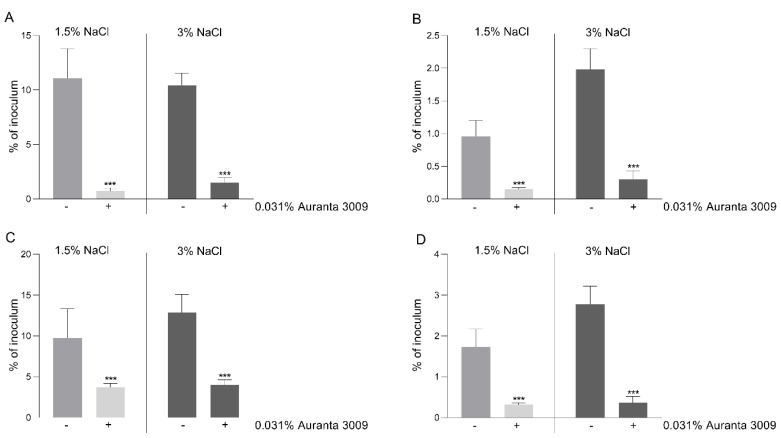
Adhesion and invasion of CHSE-214 cells by *V. parahaemolyticus* A3 and D4. The adherence (**A**) and invasion (**B**) of *V. parahaemolyticus* A3 to CHSE214 cells in the presence of Auranta 3009. Panel C shows the adherence of *V. parahaemolyticus* D4 and panel D the invasion levels of D4 to CHSE-214 cells. The results are expressed as percentages of the initial inoculum. Asterisks indicate significant differences (Student’s *t*-test ∗∗∗ *p* < 0.001). Error bars represent the standard deviation of means from three different experiments, each containing triplicate samples.

**Figure 5:**
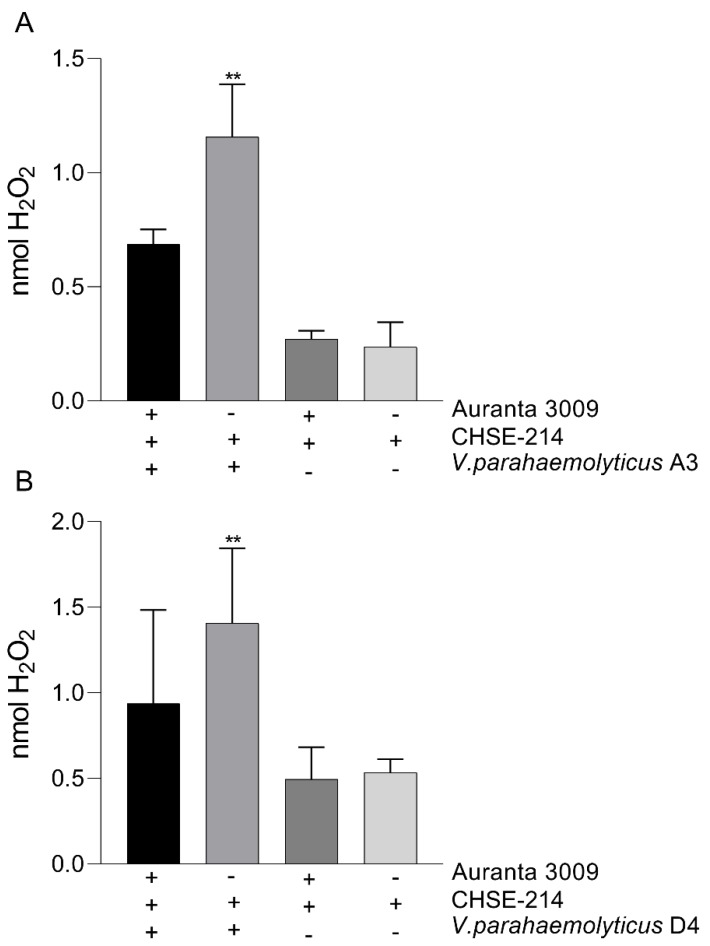
The impact of Auranta 3009 on the H_2_O_2_ production during 3 h co-culture of *Vibrio parahaemolyticus* A3 (**A**) and D4 (**B**) with CHSE-214 cells. The experiments were performed in triplicate and on three separate occasions. Student t test was used to analyse the impact of Auranta 3009 on H_2_O_2_ release (** *p* < 0.01).

In order to correlate the negative effect on motility and a possible decrease in virulence, we performed in vitro infection assays using the CHSE-214 cell line as described in Materials and Methods. The pre-treatment of *V. parahaemolyticus* A3 (Figure 4, panel A) and D4 (Figure 4, panel C) with 0.031% of Auranta 3009 significantly reduced the adherence of both strains (*p* < 0.001). The impact on bacterial internalisation was as significant as for adhesion for both strains, as indicated in Figure 5 panel B for *V. parahaemolyticus* A3 and panel D for *V. parahaemolyticus* D4 (*p* < 0.001). When the antimicrobial mixture was only present in the tissue culture media during the adhesion/invasion assay, the reductions followed the same pattern (data not shown). These results indicate that the reduced in vitro infection abilities of *V. parahaemolyticus* A3 and D4 follow a similar pattern and in concordance with the reduction in bacterial motility.

### 3.5. Challenge Tests

To estimate the efficiency of the antimicrobial mixture we performed challenge studies by infecting *P*. *vannamei* shrimps with *V. parahaemolyticus* strains A3 and D4. Our results show that at the MIC/MBC concentration of the antimicrobial (0.0625%) the lowest shrimp mortality was recorded (Table 2) for both *V. parahaemolyticus* strains A3 and D4. The challenge test confirmed the results obtained following the MIC/MBC and growth curve experiments, indicating the efficiency of the antimicrobial mixture. The percentage of mortality decreased as the concentration of the antimicrobial mixture increased. These results clearly indicated that the antimicrobial mixture has potential of protecting the shrimp cultures in vivo.

### 3.6. In vitro Epithelial H_2_O_2_ Release in Response to Treatment

We next investigated the impact of *V. parahaemolyticus* A3 and D4 infection on H_2_O_2_ release by the CHSE-214 in the presence of Auranta 3009. Our results demonstrate that the amount of H_2_O_2_ released in the media during infection is significantly reduced (*p* < 0.001) by the presence of Auranta 3009 (Figure 5). No effect was identified when the antimicrobial mixture was applied to un-infected control as the cells released H_2_O_2_ levels similar to the control. 

### 3.7. Cytotoxicity

**Figure 6:**
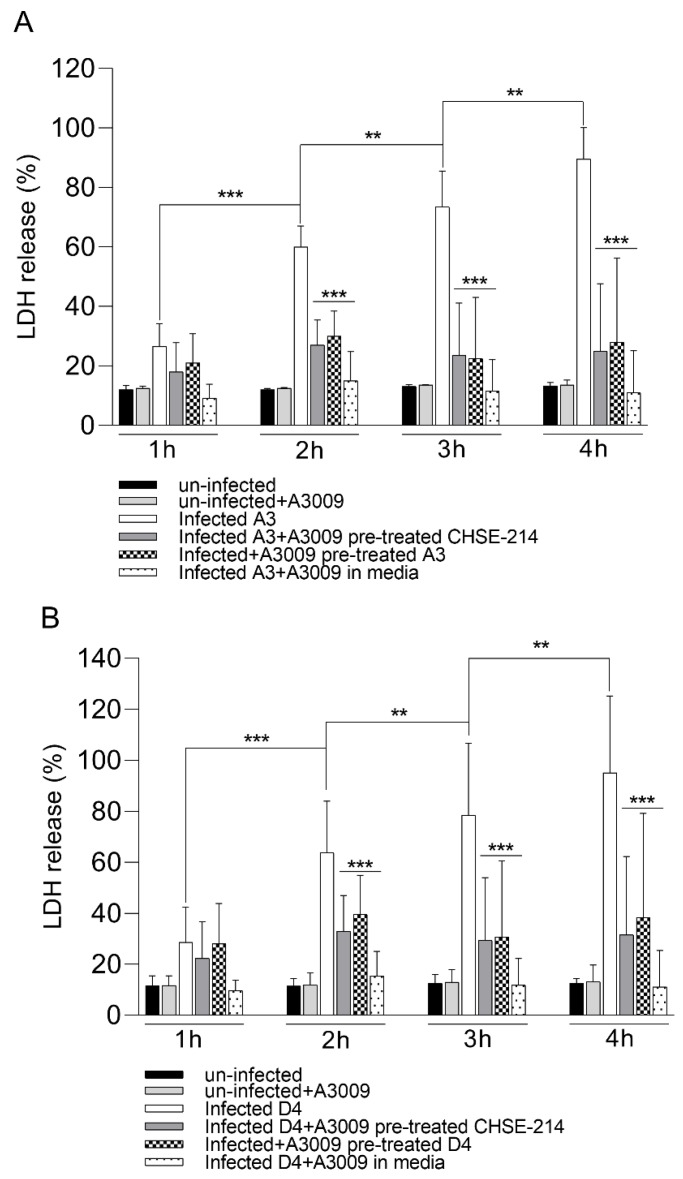
Cytotoxicity of the antimicrobial mixture to CHSE-214 cells as determined following 4 h of exposure to *V. parahaemolyticus* A3 (pannel **A**) and D4 (panel **B**) by LDH release. Data expressed as a percentage of unexposed controls of three replicates for each exposure concentration. (Student’s *t*-test ∗∗ *p* < 0.01, ∗∗∗ *p* < 0.001). Error bars represent the standard deviation of means from three different experiments performed in triplicate.

Following exposure to the 0.031% antimicrobial mixture, the infected CHSE-214 cells showed a clear reduction in cytotoxicity during infection with *V. parahaemolyticus* A3 (Figure 6, panel A) and D4 (Figure 6, panel B). The cytotoxic levels recorded for infected and un-treated cells reached over 80% at 4 h post-infection, compared to a maximum of 25% at 2 h post-infection. The highest anti-cytotoxic effect was recorded when the antimicrobial mixture was present in the culture media at all time points during infection. These results clearly show that the pre-treatment of the bacteria or of the epithelial cells prior to infection will have a significant role in keeping the cytotoxicity levels reduced (∗∗∗ *p* < 0.001). 

## 4. Discussion

Natural antimicrobials are known to be effective in reducing bacterial pathogenicity via direct [13] or indirect [26] anti-virulence activity. For example, the surface proteins involved in the virulence of *Staphylococcus aureus* are inhibited by venom peptides from being promising candidates for the treatment of related infections caused by multi-drug resistant bacteria [27] Quorum-sensing molecules originating from marine bacteria can control virulence, with gene expression being described as a novel antimicrobial strategy in aquaculture [28]. Another mechanism by which natural antimicrobials can reduce virulence refers to their involvement in bacterial physiology, as it has been shown that phenolic compounds can disrupt the expression and function of efflux pumps [29]. They can also reduce the ability of bacterial pathogens to adhere to epithelial cells in vitro (e.g., Daphnetin, a coumarin-derivative) by reducing the expression of *H. pylori* genes involved in colonisation [30]. Antimicrobial mixtures of probiotics, organic acids and essential oils were effective in reducing the devastating impact of *Vibrio* strain infections in South American farmed shrimps [31]; however, the study did not elucidate the biological mechanism responsible for the effect.

*V. parahaemolyticus* is often found in estuaries and coastal waters and because it causes severe disease is also of significant importance as a human pathogen [32]. Phage and probiotic treatments were proposed as alternative method to control infections in humans and aquaculture [23,33]. Constant and un-controlled application of increased concentrations of antibiotics in aquatic environments lead to increased resistance in the bacterial populations (e.g., *V. parahaemolyticus*), posing a threat to human and animal health and is of major concern to fish and shellfish farmers [34,35]. Early mortality syndrome is caused by infection with *V. parahaemolyticus* and strains A3 and D4 [36] were previously reported to affect shrimp farming by causing the acute hepatopancreatic necrosis disease (AHPND) [37]. *V. parahaemolyticus* strains A3 and D4 [3] acquired the type VI secretions system (T6SS), offering them a competitive advantage in survival and virulence over other bacteria in the shrimp ecosystem [7]. This development increases the need for effective control measures to prevent infection of fish and shellfish farms, especially by preventing the multiplication and virulence of *V. parahaemolyticus* in such environments [38]. 

Natural antimicrobials offer such an opportunity by being known to affect bacterial cell membranes [39]. One such example is lavender extract, which has previously been shown to reduce the ability of *V. parahaemolyticus* to produce and release its toxin [40]. The anti-virulent effect of natural antimicrobial mixtures was previously described in other pathogens e.g., *Campylobacter jejuni*, *Campylobacter coli* and Shiga Toxin Producing *E. Coli* (STEC) [13,41]. This anti-virulent effect was exhibited by decreasing the activity of major virulence factors including motility, and by reducing the in vitro and in vivo infection abilities, T6SS and biofilm formation [13,42].

According to our results, such mixtures can be used efficiently in both fresh and marine water environments, as we have observed similar results in low and high salt conditions. Similar antimicrobial mixtures have been previously used to prevent the bacterial infection of epithelial cells, both in vitro and in vivo [13], through the inactivation of virulence factors. The link between T6SS inactivation and decreased virulence has been previously shown, as it has been indicated that its presence enhances the adhesion to cultured cell monolayers [43]. This proposed hypothesis has been previously described for *C. jejuni* RC039, where the downregulation of the *hcp* gene was associated with reduced virulence both in vitro and in vivo [13]. 

In addition, *V. parahaemolyticus* A3 and D4 appear to stimulate H_2_O_2_ release from human intestinal epithelial cells, possibly by altering the redox balance within the host’s lumen or by triggering the host NADPH oxidases. Microbiota-induced alterations to the lumen redox balance could have a modulatory effect upon pathogenicity, favoring the intestinal pathogen [24]. Therefore, our results indicate that a suitable antimicrobial mixture (e.g., A3009) could be used to maintain the intestinal redox balance by restricting H_2_O_2_ release. However, the presence of hydrogen peroxide during the interaction between *V. parahaemolyticus* and the host epithelium does not seem to be critical, since it is able to significantly express catalase enzymes and induce a detoxification process [44].

## 5. Conclusions

The data presented herein describe the effect of a mixture of antimicrobials (A3009) on the virulence and pathogenicity of T6SS *V. parahaemolyticus* A3 and D4 strains. The antimicrobials reduced the ability of *V. parahaemolyticus* A3 and D4 to infect the CHSE-214 fish cell line by inhibiting the expression of the T6SS *hcp*1 and *hcp*2 genes and through the reduction of bacterial motility in both low and high salt environments. These results corroborated with the antioxidant effect, low tissue cytotoxicity and the low mortality recorded in the in vivo challenge test, suggesting that this antimicrobial mixture (A3009) can be efficiently used to improve the survival of shrimp populations when subject to *V. parahaemolyticus* infections.

## Figures and Tables

**Table 1:** MIC and MBC antimicrobial mixture concentrations required for *V. parahaemolyticus* A3 and D4 inactivation.

Specification		NaCl (%)	
		1.5	3
***V.p* A3**	MIC (%)	0.0625	0.0625
	MBC (%)	0.0625	0.0625
***V.p* D4**	MIC (%)	0.125	0.0625
	MBC (%)	0.5	0.0625

**Table 2:** Mortality of *Penaeus vannamei* after 45 h of challenge after infection with. *V. parahaemolyticus* A3 and D4.

Antimicrobial Concentration (%)	Strain	
	*V. parahaemolyticus* A3 (% of mortality)	*V. parahaemolyticus* D4 (% of mortality)
0	98.2 ± 4.3	97.3 ± 2.1
0.0625	2.8 ± 3.3	3.04 ± 1.9
0.031	11 ± 2.2	7.2 ± 4.3
0.015	18 ± 4.3	21 ± 4.4
0.007	38 ± 1.1	46 ± 5.2
